# Cooperative breeding behaviors in the Hawaiian Stilt (*Himantopus mexicanus knudseni*)

**DOI:** 10.1002/ece3.7509

**Published:** 2021-03-30

**Authors:** Arleone Dibben‐Young, Kristen C. Harmon, Arianna Lunow‐Luke, Jessica L. Idle, Dain L. Christensen, Melissa R. Price

**Affiliations:** ^1^ Ahupua‘a Natives LLC Kaunakakai HI USA; ^2^ Department of Natural Resources and Environmental Management University of Hawai‘i at Mānoa Honolulu HI USA; ^3^ Department of Ecology and Evolutionary Biology Brown University Providence RI USA

**Keywords:** chick rearing, delayed dispersal, helper‐at‐the‐nest, nest sharing, nesting success, waterbird

## Abstract

Cooperative breeding, which is commonly characterized by nonbreeding individuals that assist others with reproduction, is common in avian species. However, few accounts have been reported in Charadriiformes, particularly island‐nesting species. We present incidental observations of cooperative breeding behaviors in the Hawaiian Stilt (*Himantopus mexicanus knudseni*), an endangered subspecies of the Black‐necked Stilt (*Himantopus mexicanus*), during the 2012–2020 nesting seasons on the Hawaiian islands of O‘ahu and Moloka‘i. We describe two different behaviors that are indicative of cooperative breeding: (a) egg incubation by multiple adults; (b) helpers‐at‐the‐nest, whereby juveniles delay dispersal and reproduction to assist parents and siblings with reproduction. These observations are the first published accounts of cooperative breeding in this subspecies and merit further investigation, as cooperative breeding may improve population viability of the endangered, endemic Hawaiian Stilt.

## Graphical abstract

Cooperative breeding behaviors of Hawaiian Stilts, which have not been previously described in published literature, were observed on two Hawaiian islands. These behaviors include (a) egg incubation by multiple adults and (b) helpers‐at‐the‐nest, whereby juveniles delay dispersal and reproduction to assist parents and siblings with reproduction. Our observations merit further investigation, as cooperative breeding may improve population viability of the endangered, endemic Hawaiian Stilt.

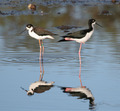

## 1 INTRODUCTION

Cooperative breeding is found throughout the world in both invertebrate and vertebrate animals (Canestrari et al., 2008; Junghanns et al., 2017; Tanaka et al., 2018). Cooperative breeding is commonly characterized by nonbreeding individuals that assist others with reproduction, potentially even delaying or foregoing their own breeding to engage in these behaviors (Brown, 1987; Cockburn, 2006; Koenig & Dickinson, 2004). Individuals may delay or forego their own dispersal and reproduction due to limitations in necessary resources for successful reproduction (Gonzalez et al., 2013; Walters et al., 1992), to inherit parental resources or resources in surrounding territories (Kokko & Ekman, 2002; Ligon & Stacey, 1991), or to increase reproductive success in related individuals, a behavior referred to as “kin‐selection” (Browning et al., 2012; Hamilton, 1964). In many cooperatively breeding avian species, nonbreeding individuals, referred to as “helpers” (Skutch, 1935), may assist the breeding pair during reproduction through their assistance with nest building, incubation, predator deterrence, and/or provisioning chicks (Koenig & Dickinson, 2016; Komdeur et al., 2017; Stacey & Koenig, 1990). These helpers are often closely related to the breeding individuals and may be previous offspring of one or both breeders (half or full siblings of the brood) or siblings of one of the breeders (uncle/aunt of the brood), therefore receiving indirect fitness benefits from this behavior (Cornwallis et al., 2009; Langen & Vehrencamp, 1999; McCarthy et al., 2001; Price et al., 2011). Although less commonly reported, cooperative breeding may also include nest sharing, by which multiple breeding individuals lay their eggs in the same nest (Barve et al., 2019; McRae, 1996); however, nests with unusually large clutch sizes may, alternatively, be due to intraspecific brood parasitism, or “egg dumping,” by unrelated females (Yom‐Tov, 1980). Drivers of nest sharing are poorly understood. However, some hypotheses include saturation of quality habitat (Barve et al., 2019) or the need for multiple females to defend a territory from challengers (Hannon et al., 1985).

In the order Charadriiformes, or waders, gulls, and auks, biparental care is most common and cooperative breeding is facultative, whereby pairs and cooperative groups coexist and dispute territories (Cockburn, 2006; Lees et al., 2013; Walters & Walters, 1980). The Hawaiian Stilt (*Himantopus mexicanus knudseni*), a member of the family Recurvirostridae (stilts and avocets), is a federally endangered, subtropical subspecies of the Black‐necked Stilt (*Himantopus mexicanus*) that breeds in wetlands across the main Hawaiian Islands. Hawaiian Stilts are pair breeders, and it is common for both the male and female to incubate eggs (Coleman, 1981). Pairs establish nesting territories, varying from 21 to 70 m^2^ in area (Coleman, 1981), that they protect and defend from potential predators and conspecifics that may destroy nests or kill chicks (Robinson et al., 2020). Hawaiian Stilts are sexually dimorphic once they reach maturity; males have metallic black plumage, while females have lighter brown glossy plumage (Coleman, 1981). Similar to other stilt species (Ackerman et al., 2014), Hawaiian Stilts typically lay a maximum of four eggs per pair (Coleman, 1981). Although there have been reports of stilt species laying more than four eggs (Coleman, 1981; Every, 1974; Hamilton, 1975), these cases have been ascribed to intraspecific brood parasitism.

The Hawaiian Stilt is relatively long‐lived, with the oldest, a male, observed 29 years after banding as an adult on Oʻahu (Reed et al., 2014). Hawaiian Stilts typically begin breeding at 2 years of age (Reed et al., 1998). Longevity and delayed reproduction are common characteristics of cooperative breeders (Brown, 1978, 1987). However, cooperative breeding behavior has not been described in published literature for this subspecies. Here, we present the first published observations of cooperative breeding behavior by juvenile and nonbreeding adult Hawaiian Stilts on the islands of O‘ahu and Moloka‘i during the 2012–2020 nesting seasons. We describe two different cooperative breeding behaviors: (a) egg incubation by multiple adults; (b) helpers‐at‐the‐nest, whereby juveniles delay dispersal and reproduction to assist parents and siblings with reproduction.

## 2 MATERIALS AND METHODS

### 2.1 Study sites

**FIGURE 1:**
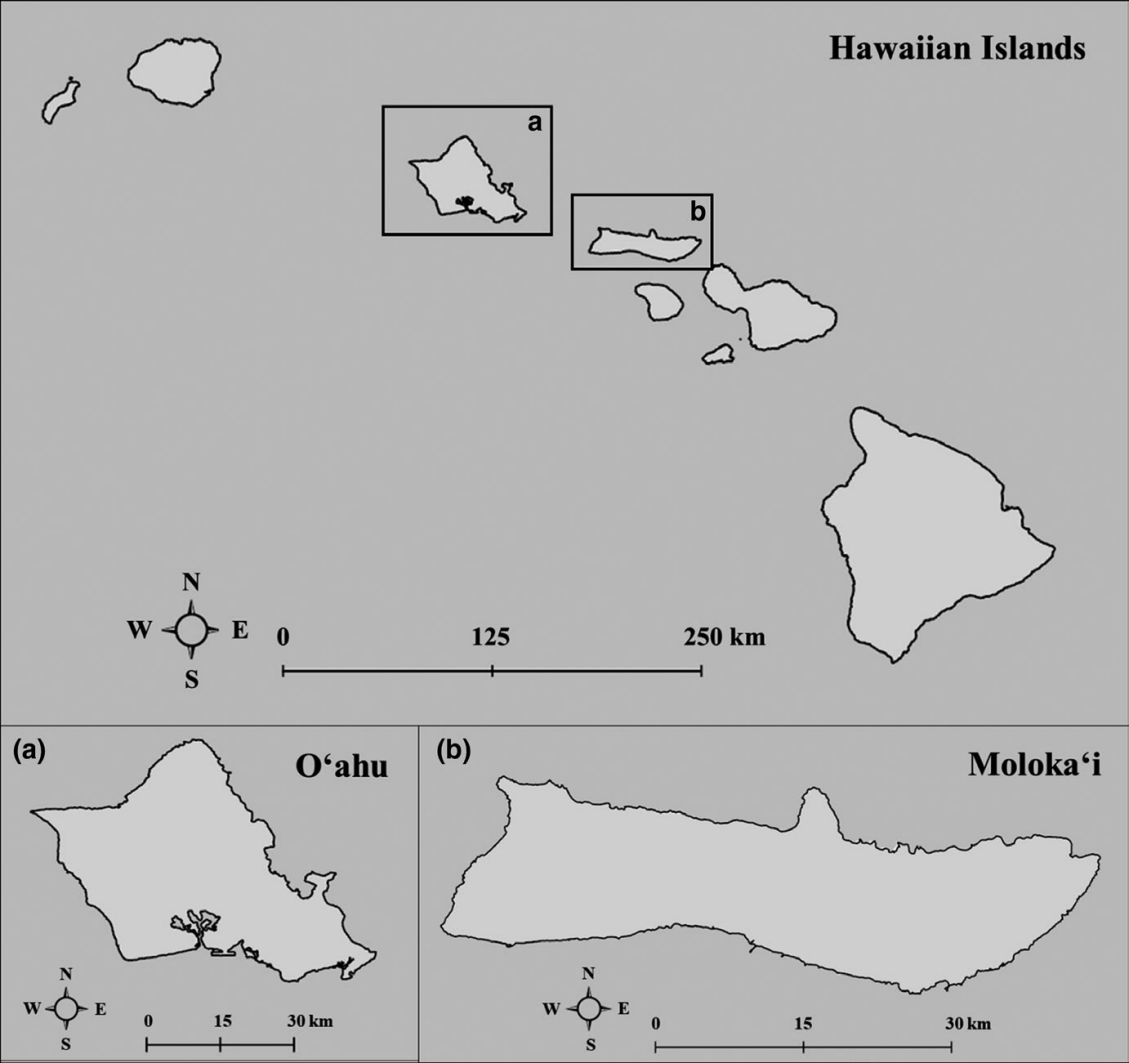
Map of (a) O‘ahu and (b) Moloka‘i within the Hawaiian Islands

Our study sites include wetlands on the Hawaiian islands of Oʻahu and Molokaʻi (Figure 1). Observations recorded in this paper are from four locations: (a) the Marine Corps Base Hawaii‐Kaneohe Bay (MCBH‐KB), located on the east side of O‘ahu; (b) Waiawa wetland, located on the west side of O‘ahu; and (c) Kōheo wetland and (d) Kaunakakai Wastewater Reclamation Facility (KWRF), both located on the south shore of Molokaʻi.

Hawaiian Stilts utilize ~60 ha of habitat at the MCBH‐KB for nesting, including the Nu‘upia Ponds Wildlife Management Area, Salvage Yard wetland, Sag Harbor wetland, Hale Koa wetland, and the Water Reclamation Facility. With the exception of the Water Reclamation Facility, water levels are not managed by MCBH‐KB staff and vary with direct rainfall and tidal fluctuations. Waiawa wetland is part of the Pearl Harbor National Wildlife Refuge and measures ~11 ha. Waiawa is composed of two ponds that receive brackish artesian well water by way of pumps managed by the U.S. Fish and Wildlife Service. Trapping of invasive mammals is conducted at both sites, including trapping of Small Indian Mongooses (*Herpestes auropunctatus*), rats (*Rattus* spp.), and feral cats (*Felis catus*).

The Kōheo wetland is a four‐ha salt marsh located on the eastern boundary of Kaunakakai. In 1989, the wetland was filled illegally for development, but in 2000, the fill was removed, and the site surrounded by T‐post and hog wire fence. The site is now utilized by students of all ages for wetland and waterbird research projects. KWRF consists of two 1 ha ponds, one of which is maintained at a water level preferred by the Hawaiian Stilt. The facility is surrounded by a chain‐link fence, and invasive mammal populations are controlled via trapping year‐round.

### 2.2 Observations from surveys

On Oʻahu in 2018, 2019, and 2020, weekly nest surveys were conducted in multiple wetlands across the island during the peak nesting season of the Hawaiian Stilt, which runs from approximately February to August (Harmon et al., 2021). All nests were monitored during the incubation period until all eggs hatched or the nest failed. Incidental observations of cooperative breeding behaviors were recorded by in‐person observers or nest cameras. On Molokaʻi, waterbird surveys were conducted at least once per week since 2012 in multiple wetlands across the island to determine waterbird densities and habitat use, as well as to relocate individuals, which were marked with a United States Geological Survey (USGS) Bird Banding Laboratory leg band and three plastic, colored leg bands. Nesting observations were incidentally recorded.

## 3 RESULTS

### 3.1 Incubation by multiple adults

**FIGURE 2:**
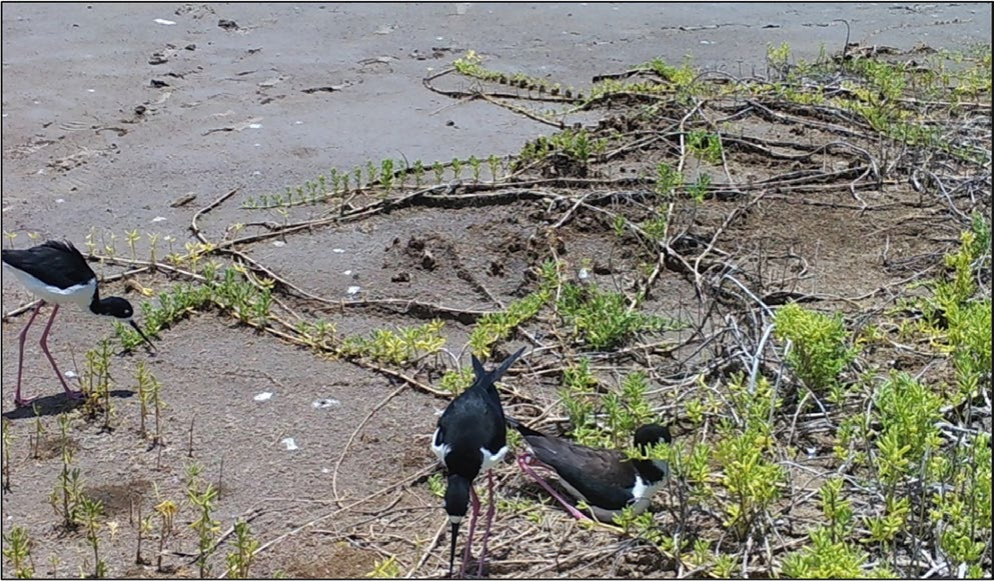
Two male Hawaiian Stilts foraging near a nest site, while one female incubates a nest with three eggs in the Waiawa wetland unit of the Pearl Harbor National Wildlife Refuge on O‘ahu on 25 May 2019. Photograph taken from a nest camera

On 25 May 2019 in Waiawa wetland, a Hawaiian Stilt nest was found with three eggs. A Bushnell trophy camera was used to monitor this nest, and using consecutive photographs, two males and one female were observed taking turns incubating the nest (Figure 2). The breeding pair was potentially assisted by a male offspring from a previous year; however, we were unable to determine the relatedness of these individuals. Our camera continued to monitor this nest for 30 days after the nest was discovered. From our observations, these eggs never hatched, although all adults continued to incubate the nest for at least 30 days.

**FIGURE 3:**
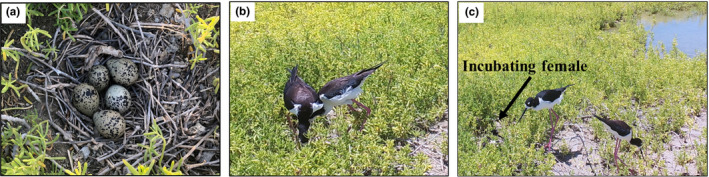
(a) Hawaiian Stilt nest found with five eggs on 25 April 2020 in wetlands in the Marine Corps Base Hawaii—Kaneohe Bay on O‘ahu. Photograph taken by JLI. (b) Two female Hawaiian Stilts standing over the nest with five eggs found in the Marine Corps Base Hawaii—Kaneohe Bay on O‘ahu. Photograph taken from a nest camera. (c) One female Hawaiian Stilt incubating the nest with five eggs, while one female and one male forage near the nest site in the Marine Corps Base Hawaii—Kaneohe Bay on O‘ahu. Photograph taken from a nest camera

On 25 April 2020, a nest with five eggs was found within MCBH‐KB on O‘ahu (Figure 3a). A Bushnell trophy camera was used to monitor this nest, and using consecutive photographs, two females were observed taking turns incubating the nest, along with one male (Figure 3b,c). From 18 May 2020 to 20 May 2020, this nest hatched three chicks, and on 21 May 2020, the adults stopped incubating the last two eggs and left the nesting area with the three chicks.

On Oʻahu, three additional nests were discovered with more than four eggs. For one nest, we only observed two adults, a male and a female, incubating the nest, and we confirmed with a nest camera that two eggs hatched, one egg was depredated, and two eggs were abandoned prior to hatching. For the other two nests, we were unable to determine how many adults were incubating the nest or the fate of the nests.

On 25 May 2017, a nest containing eight eggs was discovered at the Kōheo wetland on Moloka‘i. One adult male and two adult females were observed taking turns incubating the nest. Five eggs hatched from this nest, while three eggs were depredated, and later, two chicks were depredated; the remaining three chicks were banded, and blood collected for future determination of parental lineage. Two additional nests were discovered with five and eight eggs on Molokaʻi, but no further data were collected for these nests beyond initial identification.

### 3.2 Helpers‐at‐the‐nest

During the 2012–2015 nesting seasons at the KWRF on Moloka‘i, marked nonbreeding juveniles of Hawaiian Stilts were observed helping parents and siblings defend nesting territories. The observed individuals were marked with leg bands as chicks, and so relatedness among individuals in this population was known.

In May and June of 2012, a 1‐year‐old marked female juvenile (USGS leg band #91438709) was repeatedly observed displaying territorial behavior, such as “broken wing” and defense calls, at the nest of her marked parents, as well as at the nest of her marked grandmother. In May of 2013, that same marked female (91438709), her unmarked mate, and the female's four marked 1‐year‐old siblings (91438772, 91438719, 91438720, 91438721) were observed defending their parents' nest by displaying “broken wing” and making defense calls to a group of unrelated marked stilts. In April of 2014, these five siblings (91438709, 91438772, 91438719, 91438720, 91438721), now 3 and 2 years old, along with a marked 1‐year‐old male sibling born in 2013 (91438729), were observed displaying “broken wing” and making defense calls to a group of unrelated marked stilts at the nest of their parents. In March of 2015, a nonbreeding marked male from the 2014 brood (91438717) was observed displaying “broken wing” and making defense calls to a group of unrelated marked stilts at the nest of a marked male sibling that hatched in 2013 (91438729).

On 07 April 2013 and 25 April 2013 at the KWRF, this same family group of marked individuals, consisting of three generations, was observed protecting chicks from a group of unrelated stilts by forming a perimeter around the chicks and making defense calls.

## 4 DISCUSSION

Cooperative breeding has rarely been documented for avian species in the order Charadriiformes (Cerboncini et al., 2020; Lees et al., 2013; Walters & Walters, 1980). In this study, we present the first published observations of cooperative breeding behaviors (incubation by multiple adults and helpers‐at‐the‐nest) in multiple populations of Hawaiian Stilts on two islands more than 100 km apart. While cooperative breeding has been thought to primarily occur among related individuals (i.e., kin selection; Brown, 1987; Clutton‐Brock, 2002; Hatchwell, 2009), recent studies suggest a much broader diversity of cooperative breeding systems that involve complex alliances of relatives and nonrelatives (Riehl, 2013). It is still unclear whether the observed cooperative breeding behaviors in this study only occurred in related individuals, as we were unable to determine relations among observed individuals in some of the cases. While we did observe two female Hawaiian Stilts incubating a nest with five eggs, which could be indicative of a shared nest between two related females (McRae, 1996), all five eggs may have been laid by one female and the other female was assisting in incubation (i.e., helper‐at‐the‐nest). Alternatively, an additional egg may have been “dumped” by an unrelated female (i.e., intraspecific brood parasitism), as we were unable to determine which female laid which eggs. The breeding pair we observed in Waiawa wetland was potentially assisted by a male offspring from a previous year; however, we were unable to determine the relatedness of these individuals. Thus, the second male may have been an unrelated cobreeder (Davies, 1992).

Furthermore, limited information exists on juvenile dispersal behavior of Hawaiian Stilts (Reed et al., 1998), although we did observe delayed dispersal, coupled with helping‐at‐the‐nest, in seven marked juveniles within the KWRF on Molokaʻi. While we monitored 278 nests during our nesting study on Oʻahu (Harmon, Wehr, et al., 2021), it was often difficult to determine how many different adults participated in incubation, as most observed individuals on O‘ahu were unmarked. Thus, we were only able to report on shared incubation from three nests. Future studies that examine genetics and dispersal behavior of Hawaiian Stilts, particularly in marked populations, may help to determine how widespread cooperative breeding behaviors are in this subspecies, and whether related individuals disperse as family groups and colonize new habitats, such as recently restored wetlands.

Island systems are typically colonized by pair‐breeding species (Cockburn, 2003), but as island populations become denser and habitats more saturated, cooperative breeding is more likely to occur (Cockburn, 2003; Covas, 2012). For example, a shortage of territory openings may occur because higher quality habitats are saturated with established breeders (Arnold & Owens, 1999; Hatchwell & Komdeur, 2000). The manipulated stable conditions at the KWRF on Moloka‘i may explain the cooperatively defensive behaviors of the group of related individuals toward unrelated individuals, although group defensive behaviors are common in semi‐colonial breeders (Robinson et al., 2020). Alternatively, cooperative breeding in Hawaiian Stilts may also be a response to reduced reproductive success caused by introduced predators (van Rooij & Griffith, 2013; Sorato et al., 2012), as introduced predators are the major cause of nest failure in Hawaiian Stilts (Christensen et al., 2021; Harmon, Wehr, et al., 2021). Indeed, cooperative breeding behaviors by Hawaiian Stilts on O‘ahu were observed in areas with high nest depredation by introduced Small Indian Mongooses, rats, and feral cats. Drivers of cooperative breeding are often paradoxical. For example, both benign and harsh, as well as stable and fluctuating, environments can favor the evolution of cooperative breeding behavior (Lin et al., 2019). Thus, future studies are needed that examine both environmental and community drivers of cooperative breeding behaviors in Hawaiian Stilts.

Cooperative breeding has many potential benefits, including increased reproductive success (Downing et al., 2020) and predator avoidance (Sorato et al., 2012). However, the benefits of cooperative behavior may be outweighed by increased intraspecific competition for resources (Brouwer et al., 2020) or extremely harsh environmental conditions (Bourne et al., 2020). While nesting in close proximity may benefit reproductive success in some avian species (Downing et al., 2020), recent evidence suggests Hawaiian Stilt populations may be density‐dependent (van Rees et al., 2020), and thus, a high density of nests may lead to increased egg and chick failures in this subspecies. Furthermore, McRae (1996) found that younger female moorhens within shared nests produced fewer eggs and had lower hatching success than the older females. While some Hawaiian Stilt nests with helpers in our study were successful, others had unhatched or partially hatched nests. Further studies with larger sample sizes may allow evaluation regarding whether nests with helpers have higher success than those without helpers. Reed et al. (1998) concluded that the population viability of Hawaiian Stilts is most sensitive to increases in reproductive failure and adult mortality. However, this assessment did not account for cooperative breeding behaviors, which may improve population viability (Walters et al., 2002) and buffer extinction risk of endemic species (Mortensen & Reed, 2016). As sea‐level rise threatens coastal wetlands (Harmon, Winter, et al., 2021), a decline in available nesting habitat may provide a reproductive advantage to cooperative breeders. As clutch failure is one of the greatest threats to Hawaiian Stilts (Harmon, Wehr, et al., 2021), determining the prevalence of cooperative breeding across populations, as well as the impact of cooperative breeding on reproductive success, is critical for managing this endangered, endemic subspecies.

## CONFLICT OF INTEREST

The authors declare no conflicts of interest.

## AUTHOR CONTRIBUTIONS

**Arleone Dibben‐Young:** Conceptualization (lead); data curation (equal); funding acquisition (equal); investigation (equal); methodology (equal); project administration (equal); resources (equal); supervision (equal); validation (lead); writing‐original draft (supporting); writing‐review & editing (equal). **Kristen C. Harmon:** Conceptualization (equal); data curation (equal); funding acquisition (equal); investigation (equal); methodology (equal); visualization (lead); writing‐original draft (lead); writing‐review & editing (lead). **Arianna Lunow‐Luke:** Conceptualization (supporting); visualization (supporting); writing‐original draft (lead); writing‐review & editing (equal). **Jessica L. Idle:** Conceptualization (supporting); data curation (equal); investigation (equal); methodology (equal); validation (equal); visualization (supporting); writing‐original draft (supporting); writing‐review & editing (equal). **Dain L. Christensen:** Conceptualization (supporting); data curation (equal); investigation (equal); methodology (equal); validation (equal); visualization (supporting); writing‐original draft (supporting); writing‐review & editing (equal). **Melissa R. Price:** Conceptualization (equal); funding acquisition (equal); project administration (equal); resources (equal); supervision (equal); writing‐original draft (supporting); writing‐review & editing (equal).

## Data Availability

The data that support the findings of this study are publicly available at the University of Hawaiʻi data repository, ScholarSpace: https://scholarspace.manoa.hawaii.edu/handle/10125/75457.
